# Influence of essential amino acids on muscle mass and muscle strength in patients with cerebral stroke during early rehabilitation: protocol and rationale of a randomized clinical trial (AMINO-Stroke Study)

**DOI:** 10.1186/s12883-016-0531-5

**Published:** 2016-01-22

**Authors:** Nadja Scherbakov, Nicole Ebner, Anja Sandek, Andreas Meisel, Karl Georg Haeusler, Stephan von Haehling, Stefan D. Anker, Ulrich Dirnagl, Michael Joebges, Wolfram Doehner

**Affiliations:** Center for Stroke Research Berlin CSB, Charité - Universitätsmedizin Berlin, Berlin, Germany; German Centre for Cardiovascular Research (DZHK), partner site Berlin, Berlin, Germany; Innovative Clinical Trials, Department of Cardiology and Pneumology, University MedicineGöttingen (UMG), Göttingen, Germany; Department of Neurology, Charité - Universitätsmedizin Berlin, Berlin, Germany; Department of Neurology, Brandenburgklinik Bernau, Bernau, Germany; Department of Cardiology, Charité - Universitätsmedizin Berlin, Berlin, Germany

**Keywords:** Double blinded randomized study, Post-stroke rehabilitation, Skeletal muscle wasting, Physical performance, Essential amino acids

## Abstract

**Background:**

Patients with stroke are at a high risk for long-term handicap and disability. In the first weeks after stroke muscle wasting is observed frequently. Early post-stroke rehabilitation programs are directed to improve functional independence and physical performance. Supplementation with essential amino acids (EAAs) might prevent muscle wasting and improve rehabilitation outcome by augmenting muscle mass and muscle strength. We aim to examine this in a double blinded, randomized placebo-controlled clinical trial.

**Methods:**

Patients with ischemic or haemorrhagic stroke will be enrolled at begin of the early post-stroke rehabilitation in a parallel group interventional trial. Oral supplementation of EAAs or placebo will be given for 12 weeks in a double blinded manner. Physical and functional performance will be assessed by exercise testing before supplementation of EAAs as well as at discharge from the in-patient rehabilitation, at 12 weeks and 1 year afterwards.

**Discussion:**

This is the first randomized double-blinded placebo-controlled clinical study aiming to assess the effect of the EAAs supplementation on muscle strength, muscle function and physical performance in stroke patients during early post-stroke rehabilitation. Supplementation of EAAs could prevent muscle mass wasting and improve functional independence after stroke.

**Trial registration:**

The study is registered at the German registry for clinical trials as well as at World Health Organization (WHO; number DRKS00005577).

## Background

Long-term disability and functional dependency are the main complications after stroke. Impaired skeletal muscle innervation due to damage results in the degeneration of motor units, paresis, and immobility accompanied by skeletal muscle atrophy [1–4]. Notably, loss of muscle mass and muscle function, defined as sarcopenia, have been originally described as a phenomenon of aging [5]. However, muscle wasting observed in stroke patients is a disease-related phenomenon and the term ‘stroke-related sarcopenia’ has been suggested [1]. The aetiology of sarcopenia is multifactorial [6]. This is also true for stroke-depended sarcopenia. Several pathophysiological mechanisms including metabolic imbalance, inactivity, malnutrition, and inflammation may contribute to the reduction of muscle mass after stroke [7, 8]. Stroke-related muscle wasting is accompanied by body weight loss, neuro-hormonal activation, and a systemic shift towards catabolic over-activation [8]. In addition, activation of catabolic pathways in the skeletal muscle of the paretic and non-paretic limbs has been observed in experimental stroke [9].

Early rehabilitation has a great impact on the functional recovery after stroke. A previous study showed that 80 % of the patients achieve best functional recovery within 6 weeks after stroke, and after 12.5 weeks 95 % of all stroke patients completed their functional recovery [10]. However, one year after stroke more than 30 % of the patients remained functionally dependent [11].

Skeletal muscles play a central role in post-stroke rehabilitation. The goal of rehabilitation is to restore functional muscle capacity and to prevent long-term disability. Therefore, prevention of muscle wasting, increasing muscle strength, reactive development of novel neuromuscular junctions, and stabilisation of the catabolic-anabolic imbalance are primary aims of post-stroke rehabilitation.

Nutritional intervention may contribute to the improvement of muscle bulk and functional capacity in the early post-stroke rehabilitation. Thus, a beneficial effect of dietary supplementation of essential amino acids (EAAs) has been shown for an improvement of skeletal muscle mass and function in the elderly [12, 13]. Availability of EAAs is regarded as a limiting step in the synthesis of new muscle proteins [14]. Previously it has been shown that supplementation of EAAs in the elderly was responsible for amino acid-induced stimulation of muscle protein anabolism [15]. Therefore, an individually adjusted rehabilitation program that includes physical, functional and neuropsychological training as well as dietary supplementation of the EAA might provide the best rehabilitation outcome. Thus, in the present clinical trial the following hypotheses will be tested:

- Oral uptake of EAAs restores skeletal muscle function and physical performance;
- Oral uptake of EAAs contributes to physical independence and enhances the effectiveness of post-stroke rehabilitation;
- Availability of EAAs reduces muscle wasting after stroke.

We aim to demonstrate that nutritional supplementation with biologically limited available EAAs (l-leucine, l-lysine, l-isoleucine, l-valine, l-threonine, l-cystine, l-histidin, l-phenylalanine, l-methionine, l-tyrosin, and l-tryptophane), in synergy with individually adjusted physical training prevent muscle wasting after stroke and improve the effect of post-stroke early rehabilitation.

## Methods

### Study design

The “Influence of essential amino acids on muscle mass and muscle strength in patients with cerebral stroke during early rehabilitation (AMINO-Stroke)” study is a randomized double-blinded placebo controlled interventional clinical trial. The study is planed as a monocentric trial within the interdisciplinary trial programme of the Center for Stroke Research Berlin (CSB) and is funded by the German Federal Ministry of Education and Research (BMBF; grant number CSB 01EO1301). The study has been approved by the Brandenburg Ethics Committee for the recruiting site (S13(a)/2013) and is conducted in accordance with the Declaration of Helsinki. The study is registered at the German registry for clinical trials as well as at World Health Organization (WHO; number DRKS00005577).

### Patient eligibility

Patients admitted to the rehabilitation centre Brandenburg Klinik, Bernau, Germany within 8 weeks after ischemic or haemorrhagic stroke will be screened for the eligibility. Patients of both genders, aged 45 years or older will be informed about the study. After obtaining of written informed consent from the patients they will be randomized 1:1 to intervention and control group by a web-based online randomization procedure provided by the Pharmacy at the Charité. For inclusion and exclusion criteria see Table 1.

**Table 1:** Inclusion and exclusion criteria of the AMINO-Stroke

Inclusion criteria:
• Patient >45 years
• Patients with ischemic or haemorrhagic stroke within 8 weeks to enrolment
• Brain magnet resonance imaging or computer tomography demonstrating stroke
• Motoric disability of an upper and /or lower limb (Rivermead Motor Assessment Gross Function > 1 and <11)
• Signed informed consent
Exclusion criteria:
• Clinically significant findings on physical examination or presence of clinically significant disease that would interfere with study evaluation in the opinion of the treating physicians
• Participation in another clinical trial investigating a nutritional product
• History of intolerance or allergic response to similar nutritional products or known hypersensitivity to essential amino acids
• Clinical sighs and symptoms of infection requiring antibiotic therapy at the time of enrolment that prevent completion of trial-related assessments as judged by the investigator
• Transaminases (AST or ALT) > 3 times the upper limit of normal (ULN)
• Severe renal dysfunction or nephrotic syndrome
• Acquired immunodeficiency syndrome, HIV or Hepatitis C infection
• Current therapy with anabolic steroids or appetite stimulants
• Current immunosuppressive therapy, heart transplantation, or renal dialysis
• Life expectancy < 6 months

### Planned clinical follow-up visits

In order to evaluate an effect of the EAAs on muscle function, baseline functional assessment program will be performed at admission, followed by follow-up assessment at discharge from the in-patient rehabilitation (Table 2). All patients included into the study will receive 12 weeks either the EAAs (4 gram, 3 times a day) or placebo dissolved in a glass of water as a drinking solution.

**Table 2:** Functional assessment at baseline and during follow-up

Extended functional assessment at baseline and at 4 weeks follow-up
• Activity of daily living (modified Rankin scale, Barthel Index)
• Functional assessment scales and physical performance (Rivermead Motor Assessment Gross Function, Fugl-Meyer Scale, Functional ambulatory capacity)
• Muscle functional assessment (SPPBT, hand grip, pinch grip)
• Quality of life and nutritional status (EQ-5D, PGA)
• Blood and biomarker bank: metabolic /immunologic profile
• 24 h Holter electrocardiogram (ECG)
• Body composition by bioelectrical impendance analysis (BIA)
• Urine status
Functional assessment at 3 and 6 months follow-up
• Activity of daily living (mRS, Barthel index)
• Muscle functional assessment (SPPBT, hand grip, pinch grip)
• Quality of life and nutritional status (EQ-5D, PGA)
• Blood and biomarker bank: metabolic/immunologic profile
• 24 h Holter ECG
• Body composition by bioelectrical impedance analysis (BIA)
• Urine status

**Fig. 1:**
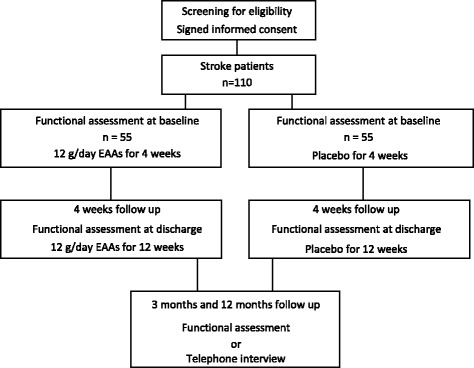
Overview of the AMINO-Stroke study

Two weeks after the enrolment a study nurse visit for evaluation of compliance of the EEAs supplementation and tolerance to the compound is planned. After discharge from the in-patient rehabilitation, two follow-up visits at 3 and at 12 months are planned. Patients will be tested for functional performance and physical independence during a 1-day ambulatory visit. Patients, who are unable to visit the study centre will be contacted by telephone and interviewed using a standardised questionnaire. The study design is summarized in Fig. 1.

### Outcomes and endpoints

#### Primary outcomes

Primary outcomes will be assessed at the 4 week follow-up visit. Primary endpoints include:

1. Physical performance according to the Rivermead motor assessment (RMA) *gross function* scale;
2. Muscle strength in a maximum hand grip strength test.

#### Secondary outcomes

Secondary endpoints include:

1. Functional outcome assessed by Barthel Index (BI);
2. Quality of life assessed according to EuroQoL (EQ)-5D (EQ-5D) questionnaire at 3 and 12 months follow up visits;
3. Muscle function and motor capabilities assessed by pinch grip strength test, motor assessment scale, Fugl-Meyer Score (motor functional domain), Functional ambulatory capacity and short physical performance battery test;
4. Changes of body composition assessed by bioelectrical impedance analysis (BIA);
5. Changes in insulin sensitivity;
6. Changes of nutritional status according to Mini Nutritional Assessment questionnaire and metabolic profile according to biochemical analyses;
7. Rate of post-stroke complications, stroke –associated infections and re-hospitalization for any reason;
8. Safety and tolerability of EEAs supplementation.

#### Physical examinations

*Rivermead motor assessment (RMA) gross function scale* comprised a series of 13 questions, and covers a range of activities from turning over in bed to hop on affected leg five times [16, 17]. *Hand grip strength* [18] *and pinch grip tests measures* muscle isometric strength. The maximum value of three measurements will be evaluated [19]. *Motor assessment scale (MAS)* includes nine items scoring from 0 to 6, and is designed to assess eight areas of motor function, balance, and muscle tone [20]. *Fugl-Meyer scale* is a system for the evaluation of motor function, balance, sensory qualities, and joint function in hemiplegic patients. This system applies a cumulative numerical score [21, 22]. *Functional Ambulation Category (FAC)* includes six categories ranging from 0 to 5 and is defined by ability of the patient to ambulate, with a lowest score 0 for “patient cannot ambulate” to 5 “patient can ambulate independently” [23].

#### Functional independency assessment

*Barthel index (BI)* will be used for the assessment of functional independence. BI includes 10 basic aspects of activities related to self-care and mobility with scores of 0–100, where the lower scores indicate greater dependency [24]. *Short physical performance battery test (SPPBT)* includes examination of ability to stand with the feet together in the side-by-side, semi-tandem, and tandem positions, time to walk 3 or 4 meters, and time to rise from the chair and return to the seated position 5 times [25].

#### Nutritional status and quality of life

*Mini Nutritional Assessment (MNA)* is a simple scale to measure nutritional status [26]. A score above 24 points identifies patients with a good nutritional status, whereas a score below 17 points reveals patients with malnutrition. *Subjective Global Assessment (SGA)* assesses nutritional status based on features of the history and physical examination [27]. *EQ-5D questionnaire* is an instrument for the generic measurements of health-related quality of life, comprising a Visual Analogue Scale for self-rating of general health and five domains evaluating mobility, self-care, usual activities, pain/discomfort, and anxiety/depression [28]. A score of 1 is the best possible general health status in each case; the dimensions may also be expressed as percentage of patients scoring from 1 to 3 (best to worst health status) or percentage of patients with ‘any’ complaint (scoring 2 or 3 in each domain) [29].

#### Body composition analysis

Body composition analysis at baseline and follow-up visits will be assessed by BIA device (BodyStat QuadScan 4000, Bodystat Limited, British Isles). The BIA device measures resistance (*R*), reactance (*Xc*) and phase angle (*φ*). The measurements will be performed in patients in supine position lying on a non-conducting surface with arms slightly abducted from the trunk and legs slightly separated, after resting at least for 20 min [30]. Surface electrodes will be placed on the dorsal surface of hand and foot of the similar side. Skeletal muscle (SM) mass will be estimated according to the equation of Baumgartner at all [31].

#### Serum biomarker assessment

Blood samples and urine status will be assessed at admission and discharge from the inpatient rehabilitation as well as at follow up visits for routine clinical parameters, inflammatory and metabolic markers, and safety analyses (liver and kidney function).

### Sample size calculation and statistical analyses

A total of 110 patients is planned to be enrolled in the study. A prospective sample size calculation has been performed based on observational data from our study centre. A sample size of 55 in each group will have 80 % power to detect a clinically relevant difference in Rivermead Motor Assessment Gross Function scale improvement between the groups of 1.5 points and a clinically relevant improvement of handgrip strength. The analysis of primary endpoints will use the principle of intention- to-treat. The carry-forward principle will be adopted for unavailable or missing data. Treatment effect will be assessed by unpaired comparison of both treatment arms for the change from baseline using the Student t-test. A significance level of α < 0.05 will be specified to indicate statistical significance. Secondary endpoints will be analyzed using standard statistical methods. For safety endpoints Chi-squared test of Fisher’s Exact test will be used for event rates (e.g., death, re-stroke, hospitalization, cardiovascular event)

## Discussion

The present AMINO Stroke study will be the first randomized double-blinded trial investigating an effect of essential amino acids (EAAs) on skeletal muscle functional capacity, muscle mass, muscle strength and physical performance in early post-stroke rehabilitation. In addition, the effect of EAAs supplementation on stroke-related quality of life, nutritional status, activities of daily life and outcome will be evaluated.

An improvement of physical and functional performance in stroke patients is expected after participating in 4-weeks rehabilitation program [32]. In the present study we expect a beneficial impact of the dietary supplementation of the EAA on skeletal muscle function and muscle mass during post-stroke rehabilitation.

The benefit of EAAs supplementation on skeletal muscle has been shown in several studies in elderly individuals. Thus, an elevated level of muscle protein synthesis was dependent on the availability of EAAs in a dose-depended manner [33]. An experimental study performing in the young adults revealed an increase of leucine incorporation into the skeletal muscle proteins under administration of EEAs as a flooding dose [34]. Ingestion of EAAs shortly after exercise enhanced muscle protein synthesis in older adults, indicating a combination of EAAs with exercising as a sufficient therapy for the treatment of muscle wasting [35]. Another study showed an improvement of lean mass, muscle functional capacity and muscle strength after 16 weeks supplementation of EAAs and arginine in healthy elderly individuals [36].

A reduction of the muscle mass might be directly depending on a limited availability of the EEAs and other nutrients [14]. Impaired feeding which results in malnutrition is frequently observed in patients with dysphagia after stroke [37]. About 50 % of stroke patients have feeding difficulties due to dysphagia [38]. In older patients reduced nutritional status might be observed already at hospital admission with the diagnosis of acute stroke [39]. Patients with lower body weight have been observed to have worse fatal and non-fatal outcome after stroke [40]. While the inverse association of overweight and improved outcome has previously been termed obesity paradox [41], the perception advanced towards an obesity paradigm, acknowledging the protective overweight in cardiovascular disease [42].

Malnutrition and sarcopenia are often reported in the rehabilitation setting [43, 44]. Therefore, we believe that supplementation of the EAAs will contribute to attenuation of muscle wasting and improve skeletal muscle recovery [45]. There are a number of methods available to assess muscle mass [46]. In the present study muscle mass will be estimated by using BIA that provides information to integrity of cell membrane and tissue interfaces, intracellular and extracellular fluid distribution, body fat and body lean mass [31, 47]. Previously, a strong correlation between muscle mass assessed by BIA and magnetic resonance imaging has been shown [48]. The prevalence of sarcopenia using BIA measurements has been repeatedly examined in elderly [49, 50], and in patients with neuromuscular or cerebrovascular disease [51, 52]. Therefore we believe that BIA is an appropriate method for muscle mass assessment.

In Germany, stroke has not been selected for chronic disease management programs so far [53]. Post-stroke rehabilitation is usually recommended to start immediately following hospital discharge. The so called early post-stroke rehabilitation phase includes about 4 weeks. Mainly, recommendations given in discharge letters from acute care hospitals are frequently focusing on medical treatments [53]. At present, post-stroke management does not include monitoring of muscle mass, muscle strength and muscle function. The European stroke guidelines do not consider post-stroke muscle wasting as a relevant complication [54]. However, recognition of this problem through the medical community might improve the long-term outcome after stroke.

## Abbreviations

EEA: Essential amino acid
BI: Barthel Index
EQ-5D: Quality of life questionnaire
RMA: Rivermead motor assessment
MAS: Motor assessment scale
FAC: Functional ambulation category
SPPBT: Short physical performance battery test
MNA: Mini nutritional assessment
SGA: Subjective global assessment
BIA: Bioelectrical impedance analysis
SM: Skeletal muscle
